# Identification of *Plasmodium falciparum* Mitochondrial Malate: Quinone Oxidoreductase Inhibitors from the Pathogen Box

**DOI:** 10.3390/genes10060471

**Published:** 2019-06-21

**Authors:** Xinying Wang, Yukiko Miyazaki, Daniel Ken Inaoka, Endah Dwi Hartuti, Yoh-Ichi Watanabe, Tomoo Shiba, Shigeharu Harada, Hiroyuki Saimoto, Jeremy Nicholas Burrows, Francisco Javier Gamo Benito, Tomoyoshi Nozaki, Kiyoshi Kita

**Affiliations:** 1 Department of Biomedical Chemistry, Graduate School of Medicine, The University of Tokyo, 7-3-1, Hongo, Bunkyo-ku, Tokyo 113-0033, Japan; xinying-wang@nagasaki-u.ac.jp (X.W.); ywatanab@m.u-tokyo.ac.jp (Y.-I.W.); nozaki@m.u-tokyo.ac.jp (T.N.); kitak@nagasaki-u.ac.jp (K.K.); 2 School of Tropical Medicine and Global Health, Nagasaki University, 1-12-4, Sakamoto, Nagasaki 852-8523, Japan; 3 Graduate School of Biomedical Sciences, Nagasaki University, 1-12-4, Sakamoto, Nagasaki 852-8523, Japan; y.miyazaki@lumc.nl (Y.M); endah.dwi08@yahoo.co.id (E.D.H.); 4 Department of Molecular Infection Dynamics, Institute of Tropical Medicine (NEKKEN), Nagasaki University, 1-12-4, Sakamoto, Nagasaki 852-8523, Japan; 5 Department of Applied Biology, Graduate School of Science Technology, Kyoto Institute of Technology, Matsugasaki, Hashikamicho, Sakyo-ku, Kyoto 606-8585, Japan; tshiba@kit.ac.jp (T.S.); harada@kit.ac.jp (S.H.); 6 Department of Chemistry and Biotechnology, Graduate School of Engineering, Tottori University, 4-101 Koyama-cho Minami, Tottori 680-8550, Japan; saimoto@chem.tottori-u.ac.jp; 7 Medicines for Malaria Venture, Route de Pré Bois 20, 1215 Geneva, Switzerland; burrowsj@mmv.org; 8 GlaxoSmithKline (GSK), Tres Cantos Medicine Development Campus, Severo Ochoa, 28760 Madrid, Spain; francisco-javier.b.gamo@gsk.com; 9 Department of Host-Defense Biochemistry, Institute of Tropical Medicine (NEKKEN), Nagasaki University, 1-12-4, Sakamoto, Nagasaki 852-8523, Japan

**Keywords:** membrane protein, mitochondria, *Plasmodium falciparum*, energy metabolism, drug target, inhibitor screening

## Abstract

Malaria is one of the three major global health threats. Drug development for malaria, especially for its most dangerous form caused by *Plasmodium falciparum*, remains an urgent task due to the emerging drug-resistant parasites. Exploration of novel antimalarial drug targets identified a trifunctional enzyme, malate quinone oxidoreductase (MQO), located in the mitochondrial inner membrane of *P. falciparum* (PfMQO). PfMQO is involved in the pathways of mitochondrial electron transport chain, tricarboxylic acid cycle, and fumarate cycle. Recent studies have shown that MQO is essential for *P. falciparum* survival in asexual stage and for the development of experiment cerebral malaria in the murine parasite *P. berghei*, providing genetic validation of MQO as a drug target. However, chemical validation of MQO, as a target, remains unexplored. In this study, we used active recombinant protein rPfMQO overexpressed in bacterial membrane fractions to screen a total of 400 compounds from the Pathogen Box, released by Medicines for Malaria Venture. The screening identified seven hit compounds targeting rPfMQO with an IC_50_ of under 5 μM. We tested the activity of hit compounds against the growth of 3D7 wildtype strain of *P. falciparum*, among which four compounds showed an IC_50_ from low to sub-micromolar concentrations, suggesting that PfMQO is indeed a potential antimalarial drug target.

## 1. Introduction

Malaria has been a constant threat and a tremendous burden to public health. In spite of effort spent in the control of malaria, the disease still caused 219 million cases and 435,000 deaths in 2017, according to a recent report issued by the World Health Organization [1]. There has been a consensus that the conventional therapies are faced by problems including the lack of an effective vaccine, reduced efficacy due to the emergence of resistant parasites, and a lack of activity towards parasites in certain life cycle stages. The fast antimalarial effect of artemisinin at its early stage of clinical use raised hopes for a reliable cure, whereas it failed to relieve the concern that there is still a long way to go before malaria elimination, as an artemisinin-resistant strain soon emerged afterwards [2]. The situation that current antimalarial drugs have been losing efficacy makes the development of new drugs an urgent task. Even though efforts on new drug discovery have never ceased, progress seems to be slow in recent decades. Since the release of Malarone^®^ (atovaquone/proguanil combination) in 2000, no new chemical class of antimalarial drug has been introduced into clinical practice [3]. In recent years, development of a high-throughput screening system largely accelerates the steps of antimalarial drug discovery. Almost 30,000 compounds identified in these screenings were reported to selectively inhibit the growth of the cultured asexual blood stage (ABS) *Plasmodium falciparum*, providing excellent starting points for the discovery of new antimalarial drugs [3,4,5]. A key strategy for malaria elimination is a combination of treatment, transmission control, and prevention using appropriate drugs. To overcome the increasing drug-resistance problem, exploration of new compounds against novel targets is of great importance. This relies on a good understanding of the parasite physiology of each life cycle stage at the molecular level [6].

The principal precondition for a compound to be used in the treatment of malaria infection is its effect on the elimination of ABS parasites [7] by targeting biological processes critical to this life cycle stage. Frequently targeted biological processes include protein synthesis in the apicoplast by clindamycin and doxycycline; host hemoglobin degradation by chloroquine and amodiaquine; endoperoxide-mediated cell damage by artemisinin; folate synthesis by sulfadoxine, pyrimethamine, and cycloguanil, and the electron transport chain (ETC) by atovaquone [8]. With the increasing problem of drug resistance, new therapies should also consider transmission control. Therefore, a drug or combination of drugs with different modes of action that target more than one biological pathway are expected to achieve adequate efficacy and delay the development of drug resistance [7]. Thus, a single compound targeting multiple biological pathways and multiple life cycle stages would be advantageous in the development of novel antimalarial drugs.

**Figure 1:**
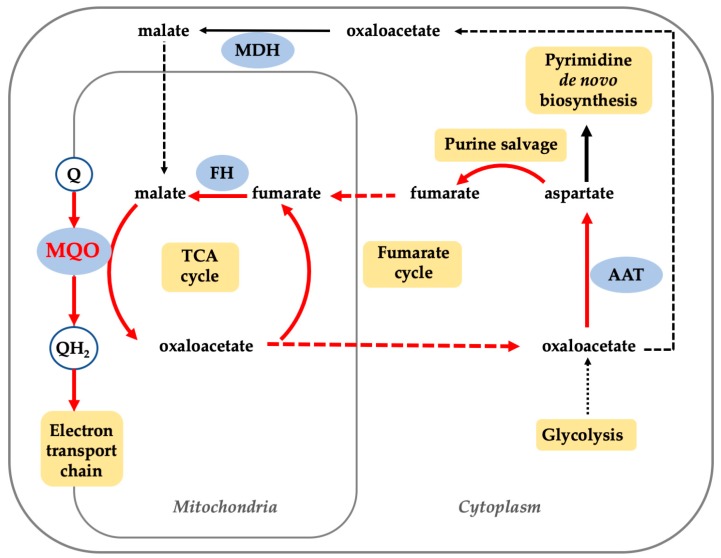
Scheme of the biological pathways in *Plasmodium falciparum* cells that PfMQO is involved with. Abbreviations, Q: ubiquinone; QH_2_: ubiquinol; FH: fumarate hydratase; AAT: aspartate aminotransferase; MDH: malate dehydrogenase.

Atovaquone is a naphthoquinone derivative that kills both the blood and liver stages of *Plasmodium* parasites [9] by targeting parasite respiration at the *bc*_1_ complex level. Although the atovaquone-resistant parasite has been reported to carry mutations in the cytochrome *b* gene coded by the mitochondrial genome, we have shown that those mutant parasites cannot spread due to an inability to develop into an oocyst inside the mosquito [10,11] indicating that mitochondrial function is important to the viability and growth of *Plasmodium* parasites at all life cycle stages. A genetic study showed that both the tricarboxylic acid (TCA) cycle and the ETC are critical for parasite development at the gametocyte and insect stages [12]. In that sense, plasmodial mitochondrial ETC dehydrogenases, which are composed by type-II NADH dehydrogenase (NDH-2) [13], succinate:quinone oxidoreductase (SQR) [14], dihydroorotate dehydrogenase (DHODH) [15], glycerol-3-phosphate dehydrogenase (G3PDH), and malate:quinone oxidoreductase (MQO) [16], are considered as potential drug targets. NDH-2, an enzyme required to reoxidize the NADH produced in TCA cycle, has been proven to be essential for *P. berghei* development at mosquito stage [17], however, the essentiality of NDH-2 in *P. falciparum* ABS was recently reported to be dispensable [18]. SQR is a member of the TCA cycle and enzymes from both *P. berghei* and *P. falciparum* have been shown to be dispensable at ABS [19,20] and at least for *P. berghei*, SQR was found to be essential for the development of oocysts [20]. Amongst the ETC dehydrogenases, DHODH, which is the rate-limiting step from pyrimidine de novo biosynthesis pathway, is the only chemogenomically validated drug target. Recent reports have demonstrated the excellent performance of DSM265, a potent and specific *P. falciparum* DHODH inhibitor, in phase Ia/b clinical trials [21]. Little is known about the essentiality of *P. falciparum* G3PDH, though it has been suggested that G3PDH is involved in gluconeogenesis and glycerol-dependent lipid metabolism pathways [22]. A recent study has shown the inability to knock out fumarate hydratase (FH) as well as MQO in the ABS of *P. falciparum* D10 strain [12]. In *P. berghei*, both FH and MQO could be genetically ablated, however, only Δ*mqo* parasite showed impaired growth phenotype and the infected mice were unable to develop cerebral malaria, indicating that MQO has an additional role in pathogenicity [23]. These results were apparently controversial to previous studies showing that the expression of a soluble type DHODH from yeast (yDHODH), which is a soluble enzyme and utilizes fumarate as electron acceptor, confers atovaquone resistance in *P. falciparum* ABS. It has long been recognized that *P. falciparum* lacks de novo purine synthesis, relying on its salvage pathway, which is indispensable for the ABS parasites [24]. Recently, MQO and FH were found to be part of the fumarate cycle, which is involved in purine salvage pathway. In this newly identified fumarate cycle, the mitochondria-derived fumarate is converted to malate and oxaloacetate through reactions catalyzed by FH and MQO, respectively. Oxaloacetate is further transported to the cytosol, where it is converted into aspartate to enter the purine salvage pathway [25], where fumarate is regenerated and transported back to mitochondria. In this way, the “fumarate cycle” is formed, linking TCA cycle and the purine salvage pathway. In these processes, MQO plays a key role by catalyzing the oxidation of malate and concomitant reduction of respiratory quinones. Hence, MQO can be considered as a trifunctional enzyme involved in the TCA cycle, ETC, and the fumarate cycle (Figure 1). In the particular case of yDHODH-3D7 phenotype, since it directly affects the fumarate cycle, the presence of yDHODH could prevent the accumulation of fumarate in the cytosol and provide a FH/MQO-independent route to perform purine salvage. Importantly, the dependence of ABS on the purine salvage pathway [24], the sexual and insect stage on the TCA cycle [12], and of the liver stage on active ETC [9,10] makes PfMQO a potential drug target for treatment, transmission-blocking, and prophylaxis of malaria. Moreover, MQO is absent in mammalian cells but it is conserved in many other pathogens, including *Toxoplasma gondii* [26], *Cryptosporidium parvum* [27], *Perkinsus marinus* [28], *Eimeria tenella* [29], *Mycobacterium tuberculosis* [29], and *Helicobacter pylori* [30]. Thus, the discovered MQO inhibitors can be used to develop drugs with novel mechanisms of action and a broad spectrum of activity. 

We have recently developed the overexpression system of PfMQO in the *Escherichia coli* membrane fraction and reported its biochemical characterization [16]. Moreover, a high-throughput screening system was also developed, which aided the identification of ferulenol, a 4-hydroxycoumarin derivative isolated from *Ferula communis*, as the first inhibitor of the MQO enzyme family [16]. However, ferulenol lacks selectivity as it appears to also inhibit *bc*_1_ complex [15]. Therefore, the primary purpose of this study is to identify potent and target-specific inhibitors which are necessary in order to chemically validate PfMQO as a drug target. In this study, we focused on 400 compounds from the Pathogen Box released by Medicines for Malaria Venture (MMV) to test their potency against both the recombinant PfMQO and the parasite ABS, with the potential to be used as tools for future chemical validation studies.

## 2. Materials and Methods

### 2.1. PfMQO Expression and Purification

The codon-optimized PfMQO gene was inserted into the pETSUMO expression system which was then used to transform BL21Star™ (DE3) chemically competent cells (ThermoFisher Scientific, Waltham, MA, USA). Recombinant PfMQO was overexpressed in the *E. coli* membrane fraction and prepared by strictly following the method established in a previous study [16]. The membrane fraction was stored in 50 mM HEPES-NaOH pH 7.6, 5 mM imidazole, 150 mM KCl, 100 µM FAD, and 50% (v/v) glycerol at −30 °C until use.

### 2.2. Screening of the Pathogen Box against Recombinant PfMQO

Four hundred drug-like compounds from the Pathogen Box were screened against recombinant PfMQO. Each compound was originally stored in dimethyl sulfoxide (DMSO) solution at 10 mM and diluted into 1 mM for screening. The screening system contained an assay mix composed of 50 mM HEPES-KOH pH 7.0, 1 mM KCN, 25 µM dUQ, 120 µM DCIP, 5 µg/mL of PfMQO-membrane fraction, and 10 µM of each compound from the Pathogen Box, and started by addition of 5 μL of 400 mM l-malate stock solution in 96-well plates. DMSO solution and 10 µM ferulenol were added in columns number 1 and 12 as negative (0% inhibition) and positive (100% inhibition) controls, respectively. PfMQO enzymatic activity was measured by SpectraMax^®^ Paradigm^®^ Multi-Mode Microplate Reader (Molecular Devices, San Jose, CA, USA) at 600 nm and 37 °C for 10 min after recording the background for 5 min. The PfMQO inhibition was determined by an end-point assay at the end of 10 min, and calculated by the remaining activity relative to negative and positive controls. Each compound inhibition was assayed in quadruplicate. Hit compounds were defined as those inhibiting more than 50% PfMQO activity at 10 µM. Z’-factor, signal-to-background ratio (S/B), signal-to-noise ratio (S/N), and coefficient of variation (CV) were calculated to estimate the quality of screening system, as previously reported [31,32]. IC**_50_** values of the hit compounds were determined under the same assay conditions for the screening, with the addition of compounds in serial dilution (10, 3, 1, 0.3, 0.1, 0.03, 0.01, 0.003, 0.001, and 0.0003 μM of final concentration). Each dilution was assayed in quadruplicate, and IC**_50_** values were calculated by applying four parameter logistic regression curve to the dose–response data using GraphPad Prism 7.0 software (GraphPad Co. Ltd., San Diego, CA, USA).

### 2.3. Plasmodium falciparum Culture

*P. falciparum* 3D7 strain was cultured in 3% hematocrit type O human red blood cell (RBC) in RPMI-1640 (Gibco, ThermoFisher Scientific, Waltham, MA, USA), supplemented with 25 mM sodium hydrogen bicarbonate, 10 µg/mL hypoxanthine, 40 µg/mL gentamicin sulfate, and 0.5% (w/v) Albumax II (Gibco, ThermoFisher Scientific, Waltham, MA, USA) under 5% O_2_ and 5% CO_2_ at 37 °C. Human RBCs were received from Japanese Red Cross Society. The experiments were performed under the guidelines of the ethical committee in The University of Tokyo (permission no. 10050) and Nagasaki University (permission no. 19). 

### 2.4. Plasmodium falciparum Growth Inhibition Assay

Parasite culture was synchronized with 5% (w/v) d-sorbitol as previously described [33]. Ring stage parasites (100 μL/well) were placed in a 96-well plate at 0.3% parasitemia. The 50% growth inhibition concentrations of compounds were determined by addition of 0.4 μL of serial dilutions (10, 3, 1, 0.3, 0.1, 0.03, and 0.01 mM) of each compound or DMSO to the wells. As a control, wells containing culture medium and RBCs alone were prepared. After 72 hours incubation, parasite growth was determined by diaphorase-coupled lactate dehydrogenase (LDH) assay, as previously described [16]. The absorbance of each well was measured at 655 nm using SpectraMax Paradigm® Multi-Mode microplate reader (Molecular Devices, San Jose, CA, USA). The inhibition rate was calculated with the absorbance of uninfected wells defined as 100% inhibition. The IC_50_ values were analyzed and calculated with GraphPad PRISM 7.0 by applying the “log(inhibitor) vs. response – Variable slope (four parameters)” in “Dose–response – inhibition” equation family.

## 3. Results

**Figure 2:**
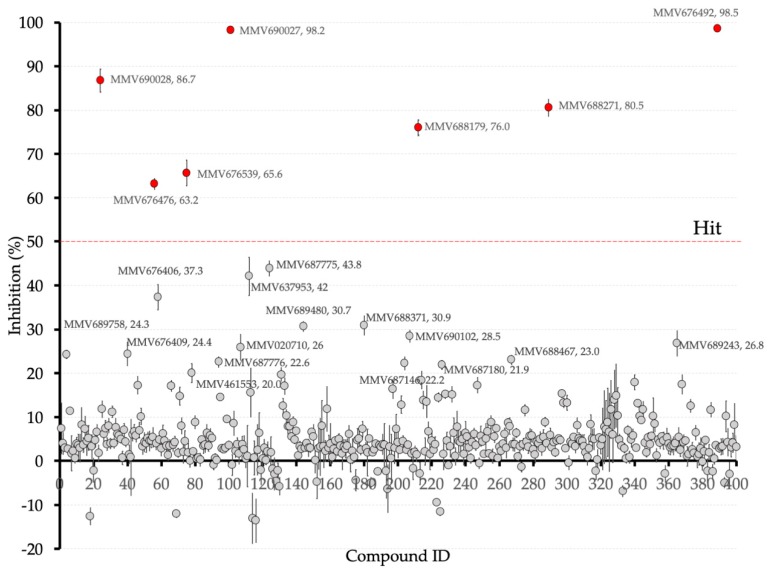
Overview of all the compounds from the Pathogen Box and their inhibition percentages against recombinant PfMQO at 10 μM.

**Figure 3:**
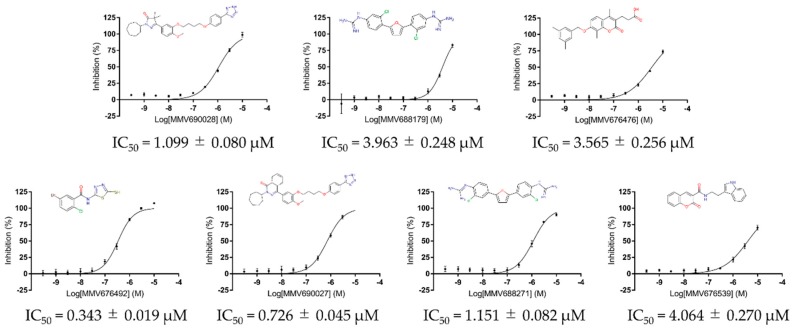
The IC_50_ against recombinant PfMQO and the chemical structure of each hit identified from the Pathogen Box.

Consistently with our previous report, PfMQO was successfully expressed in the *E. coli* membrane fraction as judged by specific PfMQO activity. After collecting the *E. coli* membrane fraction, the recombinant protein was fully active, and the specific activity increased 8.9-fold compared with homogenate, reaching 9.2 µmol/min/mg protein. With the functional protein, 400 compounds from the Pathogen Box were initially screened at 10 µM against PfMQO activity. Quality the control parameters, including Z’-factor, S/B, S/N, and CV (%) were also calculated with values of 0.905 ± 0.031, 35.694 ± 4.248, 415.968 ± 196.502, and 2.768 ± 0.951, respectively, all indicating the high quality and performance of the screening. The screening identified seven compounds inhibiting over 50% of PfMQO activity, which were defined as “hits” with a hit rate of 1.75% from the Pathogen Box (Figure 2). The potency of the seven hits (MMV676492, MMV690027, MMV690028, MMV688271, MMV676476, MMV688179 and MMV676539) was investigated and, as expected, the IC_50_ of each compound was under 5 µM, ranging from 0.343 to 4.064 µM (Figure 3). Inhibition percentages in Figure 2 and Figure 3 were calculated as average values from 4 independent experiments, with error bars representing the standard deviations.

**Figure 4:**
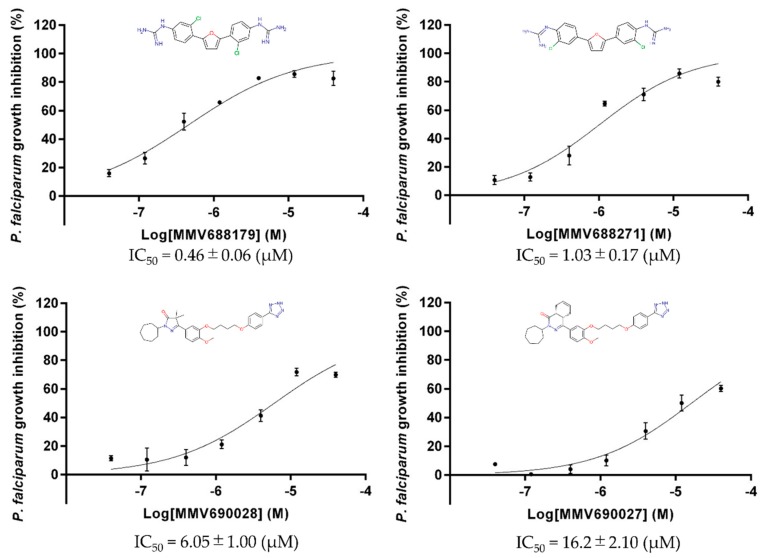
IC_50_ of each strong hit compound from the Pathogen Box which showed inhibition against the asexual blood stage (ABS) parasite.

Furthermore, inhibitory effects of the seven hit compounds on the growth of *P. falciparum* 3D7 strain in culture were evaluated by LDH assay. Among the hit compounds, four compounds (MMV688179, MMV688271, MMV690028, and MMV690027) inhibited the growth of cultured parasites, and the IC_50_ of each compound was determined as 0.46, 1.03, 6.05, and 16.2 μM, respectively (Figure 4). MMV688179 and MMV688271, as well as MMV690028 and MMV690027 pairs—which share similar backbone structures—exhibited the growth inhibition activity of the parasites at low to sub-micromolar orders. All data represent the average from three independent experiments with error bars showing the standard deviations.

## 4. Discussion

Malaria remains a life-threatening disease even though efforts have been made towards malaria elimination. Recently, programs for the discovery and development of new compounds against tropical disease have quickly expanded with investment from donor organizations and the increasing participation of the pharmaceutical industry [34]. The open source mode drug development programs, based on the concept of real-time data sharing, also provide a platform for more efficient and cheaper biomedical research [35,36]. The Pathogen Box from Medicines for Malaria Venture (MMV) is an open source chemical library, distributed free of charge to researchers upon request. It contains 400 drug-like compounds. Each compound is active against a particular pathogen and the whole box covers 13 pathogens responsible for different neglected tropical diseases. Since its release, the Pathogen Box has been tested on multiple targets reported to inhibit pathogenic fungi [37,38], helminths including *Haemonchus contortus* [39], *Schistosoma mansoni*, and *Schistosoma haematobium* [40], and apicomplexa including *Toxoplasma gondii* [41], *Cryptosporidium parvum* [42], *P. falciparum* [43], and *Neospora caninum* [44]. Importantly, screening against *P. falciparum* identified a potent inhibitor, MMV390048, that targets *Plasmodium* phosphatidylinositol 4-kinase at an IC_50_ of 28 nM [43]. 

Target-based drug discovery is another approach to increase the efficiency and productivity at an early stage of drug development. A method to produce stable recombinant target protein and the development of a high-throughput screening system are required. A recent study that established a method for expressing PfMQO in *E. coli* membrane fractions made it possible to screen the Pathogen Box to identify new inhibitors with different scaffolds against PfMQO. Of the 400 compounds tested, seven hits (≥ 50% inhibition at 10 µM) were identified with IC_50_ values below 5 µM in the first-round screening (hit rate of 1.75%), and were further tested against the growth of *P. falciparum* 3D7. Among those seven hits, four compounds showed an IC_50_ from low to sub-micromolar order. The most potent two compounds towards the growth of parasite (MMV688179 and MMV688271) with an IC_50_ of 0.46 and 1.03 µM share a backbone structure of 2-phenylguanidine groups at each side of a furan ring. The results are very consistent with a previous study where the IC_50_ values were reported to be 0.59 and 1.21 µM respectively against the ABS parasites for MMV688179 and MMV688271 [45]. It is important to note that MMV688179 was found to active against the stage IV gametocyte cells [45], and this can be possibly attributed to PfMQO inhibition due to the critical functions played by PfMQO in the TCA cycle and ETC pathways, which are essential in the gametocyte stage. These two compounds were initially synthesized and tested for their activity against *Trypanosoma cruzi* and *Leishmania donovani* [46]. They were reported to bind to DNA at the AT sites and form DNA–drug complexes resulting in the inhibition of DNA-dependent enzymes. MMV688179 shows a lower IC_50_ for parasite growth than for the enzyme, and this might be attributed to non-specific activity against DNA-dependent enzymes. MMV688271 has recently been reported to have an antifungal effect against both *Cryptococcus neoformans* and *Candida albicans*. Although those two pathogens apparently lack MQO, it may indicate a possible target amongst the mitochondrial ETC enzymes. Additionally, compounds containing phenylguanidine groups have been reported to show antibacterial activities [47]. This evidence suggests that compounds MMV688179 and MMV688271 can be potential candidates for the design of broad-spectrum antimicrobials. However, the selectivity of these two compounds needs to be improved as both compounds have HepG2 cytotoxicity effects as indicated by the CC**_20_** values of 6.8 and 13.5 µM, respectively (https://www.mmv.org/mmv-open/pathogen-box/about-pathogen-box#composition). The other two compounds MMV690028 and MMV690027, belonging to another backbone group, were less potent (IC_50_ of 6.05 and 16.2 µM respectively). MMV690028 is an inhibitor of trypanosomal phosphodiesterase B1, and has been well studied for its mode of action and structure–activity relationship [48,49]. The other three hit compounds, MMV676476, MMV676539, and MMV676492, failed to inhibit the growth of parasite cells. MMV676476 and MMV676539, belonging to the anti-tuberculosis class of compounds [36]**,** have relatively high IC_50_ values, while MMV676492 showed the strongest inhibition amongst the seven hit compounds against recombinant PfMQO. MMV676492 is a reference compound, TDR76777, from the WHO Special Program for Research and Training in Tropical Diseases (WHO/TDR), and was reported to be active against lymphatic filariasis. More studies of this compound are necessary to elucidate its lack of activity against the parasite in spite of PfMQO inhibitory activity at an IC_50_ of 0.343 µM [26]. 

## 5. Conclusions

In this study, we have developed a reliable, robust, and efficient screening method for the identification of inhibitors using a PfMQO-overexpressing *E. coli* membrane fraction. Using this method, we have successfully identified seven compounds from the Pathogen Box showing IC_50_ values below 5 μM through targeting of PfMQO, and having a different scaffold to ferulenol, the only PfMQO inhibitor reported to date. Moreover, four of these seven hit compounds showed antimalarial activity at sub-micromolar orders, suggesting that PfMQO is a potential antimalarial drug target. Future studies elucidating the target specificity of identified compounds among *P. falciparum* and mammalian ETC dehydrogenases will be conducted.
